# Reply to “The early history of the ESID registry”

**DOI:** 10.70962/jhi.20260010

**Published:** 2026-02-18

**Authors:** Markus G. Seidel

**Affiliations:** 1 Styrian Children’s Cancer Research Unit for Cancer and Inborn Errors of the Blood and Immunity in Children, Division of Pediatric Hematology-Oncology, Department of Pediatrics and Adolescent Medicine, Medical University of Graz, Graz, Austria

On behalf of the authors of the 1994–2024 registry report of the European Society for Immunodeficiencies (ESID) by Kindle and Alligon et al. (1), I would like to thank Prof. Paganelli for providing valuable historical context on the predecessors of ESID and its patient registry, namely the European Group for Immunodeficiencies (EGID) and the EGID registry (2). We—and the broader community of healthcare professionals, patient advocacy groups, and patients—are deeply indebted to the pioneering contributions of many individuals who are not listed as authors or acknowledged in this article (including Profs. M. Abedi, F. Aiuti, A. Fasth, G. Morgan, R. Paganelli, R. Seger, and others). Several of these contributors have continued to serve ESID in key leadership roles, for example, as registry chairpersons or presidents, and were, in fact, co-authors of the 2025 report. ESID and its patient registry—serving as a clinical and epidemiological database as well as a platform for numerous substudies and highly cited publications—would not be what they are today without these foundational efforts. They have grown into one of the largest medical societies and registries worldwide for patients with inborn errors of immunity, also referred to as primary immune disorders. Personally, I am grateful to Prof. Paganelli for highlighting these origins and for acknowledging the sustained efforts of the many dedicated individuals who have contributed to this achievement in his letter.
